# Karyotyping and *in situ* chromosomal localization of rDNA sites in black cumin *Bunium persicum* (Boiss) B. Fedtsch,1915 (Apiaceae)

**DOI:** 10.3897/CompCytogen.v5i4.965

**Published:** 2011-11-09

**Authors:** R. K. Chahota, Y. Mukai, H.K. Chaudhary, Naval Kishore, T.R. Sharma

**Affiliations:** 1 Department of Agricultural Biotechnology, CSK Himachal Pradesh Agricultural University, Palampur- 176 062 (HP) India; 2 Laboratory of Plant Molecular Genetics, Division of Natural Sciences, Osaka Kyoiku University, Kashiwara, Osaka 582-8582, Japan; 3 Molecular Cytogenetics and Tissue Culture Lab, Department of Crop Improvement, CSK, Himachal Pradesh Agricultural University, Palampur- 176 062 (HP) India

**Keywords:** FISH, karyotypes, *Bunium persicum*, black cumin

## Abstract

The fluorescent *in situ* hybridization (FISH) technique has been applied to somatic chromosomes in the medicinally important species, *Bunium persicum*, to elucidate its karyotypes. The bicolour FISH technique involving 18S-5.8S-26S and 5S ribosomal RNA genes as probes was used to assign physical localization and measurement of rDNA sites on homologous pairs of chromosomes. The two 18S-5.8S-26S rRNA gene sites were at the terminal regions of the short arms of the chromosomes 1 and 2 involving NOR region of chromosome 1. The 5S rDNA sites were found on subtelomeric region of the long arm of the chromosome number 5 and at interstitial regions of the short arm of chromosome 7. Based on direct visual analysis of chromosome length, morphology and position of FISH signals, a pioneer attempt has been made to construct metaphase karyotype in *Bunium persicum*, an endangered medicinal plant of North Western Himalayas.

## Introduction

Black cumin (*Bunium persicum* (Boiss) B. Fedtsch, 2n=14, is a high value medicinal and spice herb that grows as a wild plant in the forests of dry temperate and slopes of high mountainous regions of North Western Himalayas.

*In situ* hybridisation (FISH) technique has been successful for indentifying chromosome markers and physical mapping in many species of wheat, rice, lentil, and maize. Probes of repeated sequences and multigene families, including rRNA genes have become powerful tools for discerning chromosomal organization also for genetic and taxonomic relationships of agricultural plants (Lapitan et al. 1989; Mukai et al. 1991; Maluszynska and Heslop-Harrison 1991; Tsujimoto and Gill 1991). The nuclear genes encoding both 18S-5.8S-26S (45S) and 5S ribosomal RNA (rDNAs) consist of highly conserved repeat units arranged in one or more tandem arrays up to 10 000bp. In plants, the 18S-5.8S-26S rRNA genes are arrayed within the nucleolar organizing region (NOR), while the 5S rDNA is mapped outside the NOR. The independent localization makes them useful for chromosome identification (Krishnan et al. 2001). The physical mapping of repeated sequences in different crop species has also been widely reported (Irifune et al. 1995; Yamamoto et al. 1999; King et al. 2002; Lavania et al. 2005; Zhang et al. 2005). The high degree of polymorphism detected in their intergenic sequences has been extensively used for studying phylogenetic and genomic relationship among different legume species (Abirached-Darmency et al. 2005; Benabdelmouna et al. 2001). In the present study, FISH technique was applied to wild growing plants of *Bunium persicum* with objectives to elucidate physical localization of repetitive DNA sequences on metaphase chromosomes.

## Material and methods

### Plant material and preparation of cells for karyotype and FISH analysis

Primary roots from growing plants of *Bunium persicum* in pots at the Department of Crop Improvement, CSK Himachal Pradesh Agricultural University, Palampur, India were excised and pretreated in water for 16 hours at 4°C followed by fixation in ethanol: acetic acid (3:1) mixture for 5 days at room temperature. The root tips were stained in 1% aceto-carmine solution for 15 min and then squashed in 45% acetic acid. Ten well spread metaphase plates with proper chromosome contraction were analysed to prepare the standard karyotype for the species. After removing the cover glass via freezing on dry ice for 15 min, the slides were distained by immersing in 45% acetic acid for 15 min at room temperature. The air dried slides were maintained in a desiccator for at least 24 hours.

### Probe labelling

DNA probe for 45S rDNA were generated from the plasmid pTa71 containing 9kb *Eco*R1fragment of the 18S-5.8S-26S rDNA repeat sequence of *Triticum aestivum* Linnaeus, 1753 (Gerlach and Bedbrook 1979). The 18S-5.8S-26S rDNA was labelled with biotin-16-dUTP (Roche Diagnostics) by nick translation. The 5S rDNA probe was obtained from onion genomic DNA and was labelled with digoxigenin-11-dUTP (Roche Diagnostics) directly during PCR amplification according to manufacturer’s instructions. The probe mixture contained 50% (v/v) deionized formamide, 2XSSC, 10% (w/v) dextransulfate, 5 µg of salmon sperm DNA, 0.1 µg of digoxigenin-labelled 5S rRNA gene probe and 18S-5.8S-26S rRNA gene probe in final volume of 10 µl. This mixture of probes was denatured by putting hybridization mixture in boiling water for 10 min and thereafter kept on ice for 5 min.

### In situ hybridization

Chromosomal DNA on the slides was denatured in 70% deionized formamide 10% 20XSSC and 20% DDW at 70°C for 2 min then hydrated in a 70%, 95% and 100% ethanol series at -20°C for 5 min each. Slides were dried immediately with hand blower and kept for 5–10 min at room temperature. 10 µl of probe mixture were applied to each denatured preparation and covered with glass. Slides were then placed in a humid hybridization chamber at 37°C for 15 hours. After hybridization the cover glass was removed by dipping slides in 2XSSC. Slides were then washed in 2XSSC for 5 min, 50% formamide for 15 min at 40^o^ C, 2XSSC for 15 min, 1XSSC for 15 min and 4XSSC for 5 min for binding of the probe minimal homology. Few gentle shaking was done while washing in 2XSSC and 1XSSC solutions. The DNA slides were covered with parafilm after placing 65µl of antidigoxigenin rhodamine conjugate for digoxigenin labelled 5S rRNA probe and an avidine-FITC (Fluorescein isothiocyanate) conjugate for biotin labelled 18S-5.8S-26S rRNA gene probe incubated in dark at 37° C for 1 hour. The slides were then washed with 4XSSC for 10 min, 4XSSC+0.1% triton X-100 for 10 min, 4XSSC for 10 min and 2XSSC for 5 min All these steps were performed in dark and first three washing were done on orbital shaker 50rpm at room temperature. Subsequently, the slides were rinsed and mounted in a DABCO solution (1.25% DABCO in 90% glycerol) with 2.0ng/µl DAPI as a counterstain. Hybridization signals were observed under the fluorescence microscope. The observation on localization of rDNA sites at metaphase chromosomes were taken into account for final karyotyping.

## Results

### Karyotypic distribution of rDNA

Fluorescent *in situ* hybridization (FISH) was performed in order to elucidate the number and position of rDNA sites on chromosomes of the standard karyotype of *Bunium persicum*, that was revealed earlier from aceto-carmine stained chromosome plates; DAPI stained chromosomes were taken into account. *In situ* hybridization with biotin labelled probe pTa71 homologous to 18S-5.8S-26S rDNA detected as green fluorescent signals with fluorescein-conjugated avidin DN. Whereas digoxigenin-labelled 5S rDNA probe of onion detected as red fluorescent signals with rhodamine-conjugated anti-digoxigenin. The visulization of different fluorescent signals facilitated *in situ* chromosomal localization of the respective rDNA sites at varying lengths of chromosomes. It was revealed that there were 2 pairs of 18S-5.8S-26S sites in *Bunium persicum*. One pair of the homologous clusters is located on the telomeric region of the short arm of chromosome 1 and the second on the telomeric region of the short arm of chromosome 2. In chromosome 1, the rDNA site was localized at the secondary constriction region that contained the flanking terminal portion of the short arm, and a satellite, which can be visualised more easily in Figs 1c and 1d. Two pairs of 5S rDNA sites were localized in subtelomeric regions of smaller chromosomes 5 and 7. One 5S rDNA site was localized in the subtelomeric region of the long arm of chromosome 5 and the other 5S rDNA locus was observed at the interstitial region on the short arm of chromosome 7. These loci are shown in Figs 1a and 1b. Fig. 2 depicts the ideogram with the location of rDNA sites.

**Figure 1a, b.**
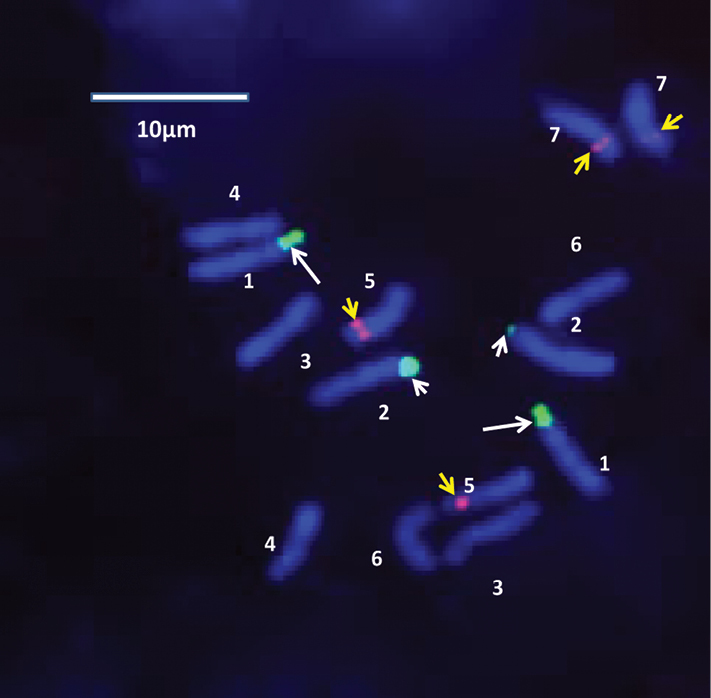
FISH on metaphase chromosomes of *Bunium persicum* using probes of 18S-5.8S-26S indicated by white arrows (NOR (white long arrows), and 5S rRNAgenes (small yellow arrows).

## Discussion

It is evident from the previous studies that rDNA loci are variable in numbers and locations in different crop (Mukai et al. 1990, 1991 in common wheat; Irifune et al. 1995 and Seo et al. 1999 in *Allium* Linnaeus, 1753 species; Abirached Darmenci 2005 in *Medicago truncatula* Gaertn., 1791; Fukui et al. 1994 in *Oryza* Linnaeus, 1753 and Lavania et al. 2005 in *Chlorophytum* Ker Gawler, 1877). Ribosomal RNA (rRNA) multigene families consist of the 18S-5.8S-26S and 5S rRNA genes. In some eukaryotes such as yeast and moss, the 5S and 18S-5.8S-26S rRNA genes are in juxtaposition in the same locus, whereas in other eukaryotes, they are organized as families of tandemly repeated units located at one or a few chromosomal sites (Flavell 1986) and may be unlinked on the same chromosome arm or located on different chromosomes (Mukai et al. 1990). Visualization of the 5S and 18S-5.5S-26S rRNA genes by FISH has provided a number of chromosomal markers to elucidate the chromosomal evolution and species interrelationships (Mukai 2005). In the past decades, FISH studies have been conducted in numerous plant species to elucidate the number and localization of rDNA sites. It has been observed that most of the diploid plants have two sites (i.e. a single locus) of both 5S and 18S-5.8S-26S rDNA (Mishima et al. 2002), although some diploids may have multiple sites (Fukui et al. 1994; Badaeva et al. 1996; De Bustos et al. 1996; Raina and Mukai 1999; Lavania et al. 2005). Prokopowich et al. (2003), based on exhaustive analysis on the rDNA copy number and genome size in large number of animal and plant taxa, have suggested a strong positive correlation between genome size and rDNA copy number. This helped us to understand that ribosomes would increase as genome size increases if the relative proportion of protein-coding genes remains constant.

In *Bunium persicum* all chromosomes were arranged according to the length, morphology and chromosomal markers (18S-5.8S-26S and 5S rDNA), with the largest chromosome designated as the chromosome 1 and smallest as the chromosome 7 (Fig. 2). The majority of the chromosomes were metacentric making identification of homologous pairs difficult as the size gradient and morphology of chromosomes 5 and 7 and chromosomes 4 and 6 was conspicuous. Identification of long and short arms on which rDNA loci were located was achieved by comparing the same photographs taken before and after *in situ* hybridization (Figs 1c, d). Slight variation in the long arm of chromosomes 4 and 6 helped us to identify homologous pair of each chromosome. The position of two pairs of 5S rDNA sites at different arms was used to identify homologous pairs of chromosome 5 and 7. On the chromosome 5, the 5S rDNA site was located on long arm at subtelomeric region, whereas it was at the interstitial region of the short arm of chromosome 7. Due to small variation in 5S rDNA sites in chromosomes 5 and 7, FISH analysis was carried out at prophase stage to identify the exact location of 5S rDNA sites in these chromosomes (Fig. 1c). The present study revealed that there were two sites for 18S-5.8S-26S rDNA loci each at telomeric regions on chromosomes 1 and 2. Therefore, the identification of chromosomes 1 and 2 was ascertained by visualizing 18S-5.8S-26S rrDNA signals. The 18S-5.8S-26S rDNA signals were over a longer distance on chromosome 1, indicating the presence of secondary constriction (NOR) and a satellite of the chromosome. Some authors have reported that the 18S-5.8S-26S rRNA multigene family, as a component of the nucleolar organizing region (NOR) which is strongly hybridized to the secondary constriction and satellite (Mukai et al. 1991; Hizume 1994; Castilho and Heslop-Harrison 1995). Although the satellite identification by conventional staining is very difficult because of its small and fragile constriction site, FISH signal allowed the identification of chromosomes with similar morphology. Therefore, the chromosomes 1 and 2 despite having the same size and presence of 18S-5.8S-26S rDNA loci at terminal regions could be discriminated by the long FISH signals on chromosome 1. The bi-or multicolour FISH technique, using rRNA multigene families and other detectable DNA sequences as probes will be useful for determining the marker chromosomes that are similar in size and morphology among species. The present study revealed that chromosomes 5 and 7 were marker chromosomes for 5S rRNA gene and that chromosomes 1 and 2 were marker chromosomes for18S-5.8S-26S rRNA gene. The establishment of the karyotype for *Bunium persicum* may allow the assignment of linkage groups by FISH. This will help to undertake further cytogenetic studies and physical mapping of the loci that can act as landmarks source for the development of genetic map in this plant.

**Figure 1c.**
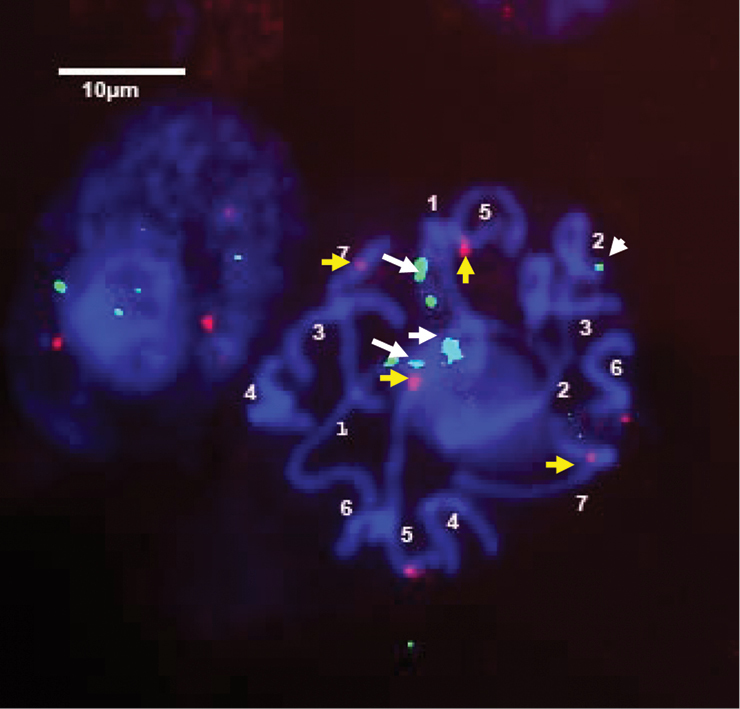
FISH on Prophase chromosomes of *Bunium* using probes of 18S-5.8S-26S indicated by white arrows (NOR (white long arrows), and 5S rRNAgenes (small yellow arrows).

**Figure 1d.**
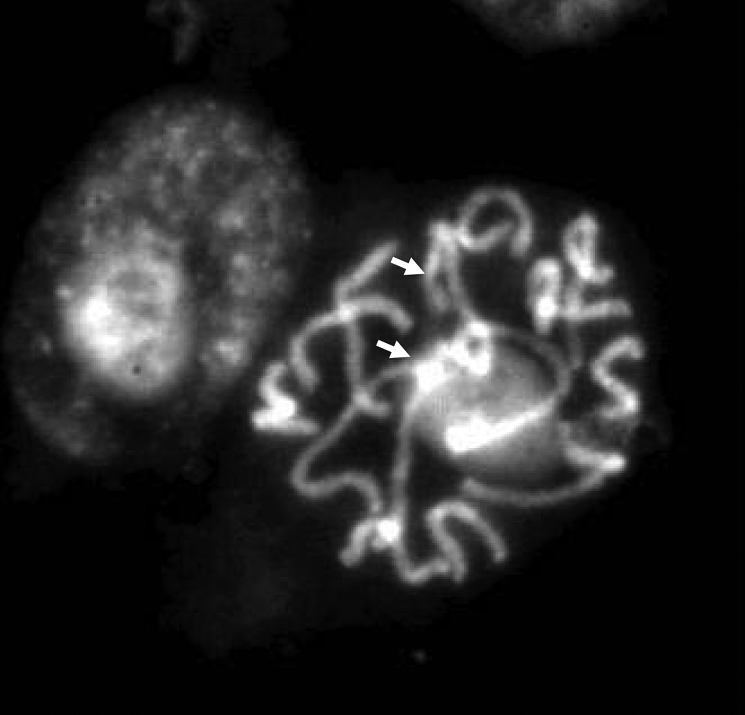
Prophase chromosome of *Bunium persicum* with DAPI to visualise the NOR regions (white arrows).

**Figure 2.**
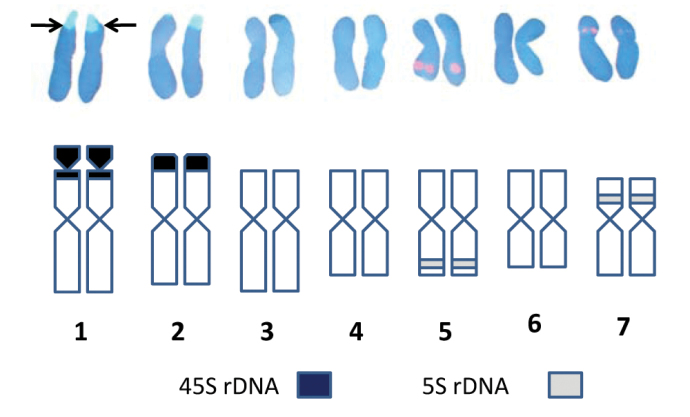
Karyo-idiograms of *Bunium persicum* showing FISH based localization of two rDNA sites (18S-5.8S-26S and 5 S rDNA) on somatic chromosomes of *Bunium persicum*.
